# Outcome of patients with heart failure after transcatheter aortic valve implantation

**DOI:** 10.1371/journal.pone.0225473

**Published:** 2019-11-26

**Authors:** Ulrich Fischer-Rasokat, Matthias Renker, Christoph Liebetrau, Maren Weferling, Andreas Rolf, Mirko Doss, Helge Möllmann, Thomas Walther, Christian W. Hamm, Won-Keun Kim

**Affiliations:** 1 Department of Cardiology, Kerckhoff Heart Center, Bad Nauheim, Germany; 2 Department of Cardiology and Angiology, Medical Clinic I, University Hospital of Giessen, Giessen, Germany; 3 Department of Cardiac Surgery, Kerckhoff Heart Center, Bad Nauheim, Germany; 4 German Centre for Cardiovascular Research (DZHK), Partner Site RheinMain, Bad Nauheim, Germany; 5 Department of Cardiology, Medical Clinic I, St. Johannes Hospital, Dortmund, Germany; 6 Department of Cardiac, Thoracic and Thoracic Vascular Surgery, University Hospital of the Goethe University, Frankfurt/Main, Germany; University of Bern, University Hospital Bern, SWITZERLAND

## Abstract

**Aims:**

Patients with aortic stenosis (AS) may have concomitant heart failure (HF) that determines prognosis despite successful transcatheter aortic valve implantation (TAVI). We compared outcomes of TAVI patients with low stroke volume index (SVI) ≤35 ml/m^2^ body surface area in different HF classes.

**Methods and results:**

Patients treated by transfemoral TAVI at our center (n = 1822) were classified as 1) ‘HF with preserved ejection fraction (EF)’ (HFpEF, EF ≥50%), 2) ‘HF with mid-range EF’ (HFmrEF, EF 40–49%), or 3) ‘HF with reduced EF’ (HFrEF, EF <40%). Patients with SVI >35 ml/m^2^ served as controls. The prevalence of cardiovascular disease and symptoms increased stepwise from controls (n = 968) to patients with HFpEF (n = 591), HFmrEF (n = 97), and HFrEF (n = 166). Mortality tended to be highest in HFrEF patients 30 days post-procedure, and it became significant after one year: 10.2% (controls), 13.5% (HFpEF), 13.4% (HFmrEF), and 23.5% (HFrEF). However, symptomatic improvement in survivors of all groups was achieved in the majority of patients without differences among groups.

**Conclusions:**

Patients with AS and HF benefit from TAVI with respect to symptom alleviation. TAVI in patients with HFpEF and HFmrEF led to an identical, favorable post-procedural prognosis that was significantly better than that of patients with HFrEF, which remains a high-risk population.

## Introduction

In specific high-risk patient populations with aortic stenosis (AS), transcatheter aortic valve implantation (TAVI) has only a minor impact on mortality and has even been considered to be a futile treatment with respect to symptom alleviation [1–3]. Efforts to identify these patients and the underlying pathological mechanisms have yielded several parameters that are independently associated with poor outcome after TAVI: low transvalvular gradients (MPG), low stroke volume index (SVI), low ejection fraction (EF), or any combination of these [4–8]. All of these clinical findings can be subsumed into the diagnosis of heart failure (HF), given that there is an almost complete overlap of functional, echocardiographic, hemodynamic, and biochemical measurements between patients with symptomatic AS or HF. The concept of concomitant HF in patients with AS is supported by findings that patients with HF and AS have a similar shift in myocardial substrate metabolism [9] and that there are parallels in invasive left ventricular (LV) hemodynamics between the two conditions [10]. Furthermore, the LV displays structural and functional impairments after TAVI that persist after correction of afterload: left ventricular hypertrophy (LVH) has been demonstrated to persist above the upper limit of normal after aortic valve replacement and is associated with impaired long-term survival [11]. Likewise, elevated levels of natriuretic peptides show only a modest decrease after TAVI [12] or may even persist at a high level for many years [13]. Finally, recent clinical studies [14, 15] suggest that the concomitant cardiomyopathy in patients with AS is the primary driver of mortality that evades effective treatment by TAVI and determines further prognosis. It remains, however, challenging to diagnose concomitant HF in patients with AS, which is reflected in the plethora of parameters used for this definition [16, 17] and a general uncertainty about cut-off values for EF [14, 15].

Our aims in undertaking this study were to compare outcomes of patients in different HF classes after correction of increased afterload by TAVI.

## Methods

### Study design, setting, and participants

In this retrospective, observational study, we analyzed the outcome of patients with symptomatic AS and concomitant HF after TAVI. All data derived from a TAVI registry at a single center. Since 2011, patients who underwent TAVI for symptomatic AS were consecutively included as a result of the local heart team decision at our center. Follow-up visits were scheduled at 30 days and one year post-TAVI. The data were collected in a standardized and anonymized format. The approval for this study was obtained from the ethics committee of the Justus-Liebig University Giessen. Due to the retrospective nature of this study a waiver of written informed consent was issued by the ethics committee.

**Fig 1:**
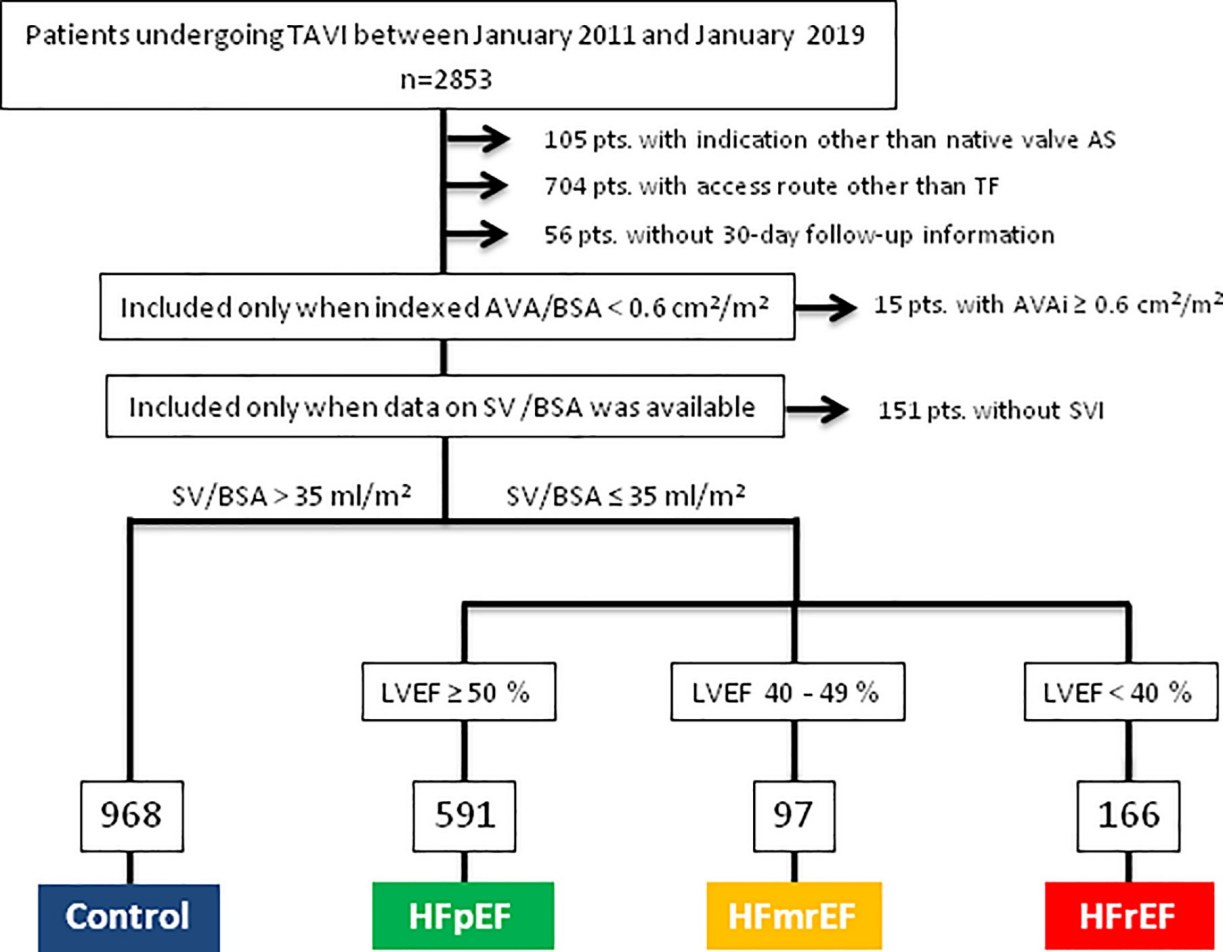
Flow chart illustrating the four groups derived from the entire patient population. AS = aortic stenosis; AVA = aortic valve area; AVAi = AVA index; BSA = body surface area; MPG = mean pressure gradient; SV = stroke volume; LVEF = left ventricular ejection fraction; HFpEF = heart failure with preserved ejection fraction; HFmrEF = heart failure with mid-range ejection fraction; HFrEF = heart failure with reduced ejection fraction; TF = transfemoral.

The flowchart for the creation of study groups and sizes is given in Fig 1. Patients were diagnosed as having concomitant HF based on two criteria: a) symptoms and signs and b) low SVI. As it was impossible to attribute symptoms and signs to either AS or HF with certainty, we defined patients with symptomatic AS to have concomitant HF only if SVI was low (≤35 ml/m^2^). Those patients with HF were further categorized based on the EF as ‘HF with preserved ejection fraction (EF)’ (HFpEF, EF ≥50%), 2) ‘HF with mid-range EF’ (HFmrEF, EF 40–49%), or 3) ‘HF with reduced EF’ (HFrEF, EF <40%). Patients with symptomatic AS and SVI >35 ml/m^2^ were classified as controls.

### Outcome variables

The primary endpoint was death from any cause. Death of unknown origin was classified as cardiovascular. Patients with follow-up time longer than one year were censored as alive after 365 days.

### Echocardiographic measurements

Echocardiographic exams were scheduled before TAVI, before discharge from hospital, and at 30 day follow-up. SV was determined at the LV outflow tract by multiplying area by the systolic velocity integral. Aortic valve area (AVA) was calculated according to the continuity equation [18]. Patients with low-gradient AS and calculated SVI <35 ml/m^2^ were examined by transesophageal echocardiography to confirm AVAi <0.6 cm^2^/m^2^ by planimetry. EF was estimated visually. LV mass was calculated by the linear method [19]. LVH was defined as LV mass >95 g/m^2^ in women and >115 g/m^2^ in men [19].

### Measurement of aortic valve calcification

Noncontrast multidetector computed tomography was used for measurement of aortic valve calcification (AVC). AVC density (AVCd) was calculated as AVC indexed to the cross-sectional area of the aortic annulus, which was calculated from the diameter of the left ventricular outflow tract measured by echocardiography according to Clavel et al. [20]. Anatomically severe AS according to the sex-specific cut-off values were defined for AVC scores ≥1200 AU in women and ≥2000 AU in men [18] and for AVCd values ≥300 AU/cm^2^ in women and ≥500 AU/cm^2^ in men [20].

### Statistical analysis

Data are median and interquartile range (IQR). Categorical variables are presented as numbers (percentages). Continuous values were compared by the Mann-Whitney or Kruskal-Wallis test and categorical variables by the χ^2^ test. Survival curves were constructed using Kaplan-Meier estimates and were compared by the log-rank test. Multivariate Cox regression analyses were performed with mortality as the outcome variable considering the following baseline parameters: sex, BMI, GFR, NYHA class III or IV, prior cardiac decompensation, DM, COPD, STS score, prior myocardial infarction, atrial fibrillation, prior ICD implantation, EF, LV myocardial infarction, SVI, MPG, mitral regurgitation ≥II°, tricuspid regurgitation ≥II°, SPAP, severe AVCd and the use of a balloon-expandable valve. Univariate predictors with P≤0.1 were entered into multivariate Cox regression analysis. The HF classification was not entered into the analysis because of high co-linearity. All statistical analyses were performed using the SPSS statistical package version 24 (IBM Corp., Armonk, NY, USA).

## Results

### Patient characteristics

A total of 591 patients with HFpEF, 97 patients with HFmrEF, 166 patients with HFrEF, and 968 patients in the control group were identified (Table 1). There was a distinct, stepwise increase in symptom severity comparing the controls with HFpEF, HFmrEF, and HFrEF with respect to the percentage of patients with severely reduced NYHA functional status and with respect to the percentage of patients with prior episodes of cardiac decompensation; the levels of NT-proBNP increased in parallel. Interestingly, more than 50% of all patients with HF but only 30% of the control patients presented with atrial fibrillation. The predicted perioperative mortality according to the STS score varied between 4.1 (2.7–5.6) and 6.0 (4.0–8.5) % between patients in the control, HFpEF, HFmrEF, and HFrEF groups (p<0.001).

**Table 1:** Patient characteristics of entire study population.

	Control	HFpEF	HFmrEF	HFrEF	p
	n = 968	n = 591	n = 97	n = 166	overall
**Demographic data**					
Female	549 (56.7)	358 (60.6)	39 (40.2)	53 (31.9)	<0.001
Age, y	82 (79–85)	83 (79–86)	82 (79–85)	80 (76–84)	<0.001
BMI, kg/m^2^	26.6 (23.9–29.5)	27.2 (24.5–31.2)	27.8 (24.9–32.0)	25.9 (23.5–29.6)	<0.001
GFR, ml/min/1.73 m^2^	70 (48–90)	63 (45–83)	59 (42–75)	54 (40–69)	<0.001
Dialysis	17 (1.8)	9 (1.5)	2 (2.1)	7 (4.2)	0.152
NYHA class III / IV	696 (71.9) / 73 (7.5)	422 (71.4) / 62 (10.5)	69 (71.1) / 16 (16.5)	111 (66.9) / 35 (21.1)	<0.001
Prior cardiac decompensation	232 (24.0)	181 (30.6)	46 (47.4)	94 (56.6)	<0.001
NT-proBNP, ng/L	1331 (667–2557)	2061 (952–4442)	2364 (1105–7861)	6043 (3287–8071)	<0.001
	*n = 96*	*n = 69*	*n = 10*	*n = 33*	
**Risk factors**					
Arterial hypertension	896 (92.6)	544 (92.0)	92 (94.8)	146 (88.0)	0.157
Diabetes mellitus	284 (29.3)	202 (34.2)	38 (39.2)	74 (44.6)	<0.001
Dyslipidemia	366 (37.8)	216 (36.5)	34 (35.1)	62 (37.3)	0.929
COPD	198 (20.5)	117 (19.8)	10 (10.3)	22 (13.3)	0.020
STS Score	4.1 (2.7–5.6)	4.5 (2.9–6.5)	4.6 (3.1–7.5)	6.0 (4.0–8.5)	<0.001
**Cardiovascular disease**					
CAD	558 (57.6)	340 (57.5)	66 (68.0)	112 (67.5)	0.024
CABG	98 (10.1)	62 (10.5)	19 (19.6)	36 (21.7)	<0.001
Prior myocardial infarction	96 (9.9)	61 (10.3)	20 (20.6)	46 (27.7)	<0.001
Atrial fibrillation	293 (30.3)	325 (55.1)	49 (50.5)	90 (54.2)	<0.001
PM / ICD	76 (7.9) / 12 (1.2)	77 (13.0) / 2 (0.3)	17 (17.5) / 3 (3.1)	14 (8.4) / 19 (11.4)	<0.001
Prior stroke	120 (12.4)	89 (15.1)	12 (12.4)	18 (10.8)	0.362
Peripheral artery disease	102 (10.5)	66 (11.2)	10 (10.3)	25 (15.1)	0.392

Data shown as number (%) or median value (interquartal range). Abbreviations: HFpEF: heart failure with preserved ejection fraction; HFmrEF: heart failure with mid-range ejection fraction; HFrEF: heart failure with reduced ejection fraction; BMI = body mass index; GFR = glomerular filtration rate (estimated); NYHA = New York Heart Association; COPD = chronic obstructive pulmonary disease; STS = Society of Thoracic Surgeons; CAD = coronary artery disease; CABG = coronary artery bypass grafting; PM/ICD = pacemaker/implantable cardioverter-defibrillator

Median EF was normal in controls and HFpEF patients and moderately and severely reduced in patients with HFmrEF and HFrEF, respectively (p<0.001; Table 2). 74% of patients with HFpEF and 86% with HFmrEF fulfilled the criteria for LVH. SVI declined within the HF classes from HFpEF (30[26–32]ml/m^2^), to HFmrEF (27[23–31]ml/m^2^) to HFrEF (24[19–29]ml/m^2^), in parallel with the transvalvular MPG (42[30–51]mmHg vs. 34[24–47]mmHg vs. 24[19–29]mmHg; p< 0.001). Conversely, the prevalence of more-than-moderate mitral or tricuspid valve regurgitation progressively increased from controls to HFrEF patients. Anatomically severe AS in HF patients with low SVI was confirmed in more than 70% and in more than 76% according to the criteria for AVC and AVCd, respectively.

**Table 2:** Doppler echocardiographic and MDCT data.

	Control	HFpEF	HFmrEF	HFrEF	p
	n = 968	n = 591	n = 97	n = 166	overall
**Echocardiographic data**					
Ejection fraction, %	65 (60–65)	65 (60–65)	45 (40–45)	30 (25–34)	<0.001
LV mass index, g/m^2^	130 (111–154)	123 (103–142)	145 (122–161)	143 (118–175)	<0.001
LV hypertrophy	709 / 874 (81.1)	380 / 515 (73.8)	69 / 80 (86.3)	122 / 146 (83.6)	0.002
SVI, ml/m^2^	42 (38–47)	30 (26–32)	27 (23–31)	24 (19–29)	<0.001
MPG, mmHg	45 (37–55)	42 (30–51)	34 (24–47)	24 (17–37)	<0.001
AVAi, cm^2^/m^2^	0.39 (0.34–0.45)	0.30 (0.25–0.36)	0.32 (0.26–0.38)	0.35 (0.27–0.42)	<0.001
MR ≥ 2+	80 (8.3)	87 (14.7)	26 (26.8)	42 (25.3)	<0.001
TR ≥ 2+	65 (6.7)	76 (12.9)	20 (20.6)	40 (24.1)	<0.001
MR ≥ 2+ & TR ≥ 2+	25 (2.6)	29 (4.9)	9 (9.3)	16 (9.6)	<0.001
SPAP, mmHg	42 (35–53)	44 (36–58)	48 (37–62)	48 (38–55)	0.003
**MDCT data**					
AVC, AU	2544 (1755–3509)	2470 (1636–3472)	2790 (1704–3984)	2258 (1638–3264)	0.132
Meeting criteria for severe AS	812 / 954 (85.1)	481 / 578 (83.2)	72 / 97 (74.2)	116 (164 (70.7)	<0.001
AVCd, AU/cm^2^	798 (569–1097)	840 (580–1219)	768 (483–1340)	714 (456–1014)	0.001
Meeting criteria for severe AS	873 / 953 (91.6)	532 / 574 (92.7)	80 / 97 (82.5)	126 / 164 (76.8)	<0.001

Data shown as number (%) or median value (interquartal range). Abbreviations: HFpEF: heart failure with preserved ejection fraction; HFmrEF: heart failure with mid-range ejection fraction; HFrEF: heart failure with reduced ejection fraction; LV = left ventricular; SVI = stroke volume index; MPG = transvalvular mean pressure gradient; AVAi = aortic valve area index; MR = mitral regurgitation; TR = tricuspid regurgitation; SPAP = systolic pulmonary artery pressure; MDCT = multidetector computed tomography; AVC = aortic valve calcification; AVCd = AVC density; AU = arbitrary units

### Procedural data

More balloon-expandable valves were used in patients in the HFrEF (44.6%) and HFmrEF (45.8%) groups than in the control (33.6%) and HFpEF (38.1%) groups (p = 0.006) (Table 3). Procedural time, the amount of contrast agent, the device success according to the Valve Academic Research Consortium-2 (VARC) criteria, and the percentage of residual aortic regurgitation ≥II° were not different between groups. The post-procedural median MPG showed a small but significant decrease from control patients (10[8–13]mmHg) progressively through the HF classes to patients with HFrEF (8[6–11]mmHg, p<0.001).

**Table 3:** Procedural data.

	Control	HFpEF	HFmrEF	HFrEF	p
	n = 968	n = 591	n = 97	n = 166	overall
Transfemoral access	968 (100)	591 (100)	97 (100)	166 (100)	ns
Balloon-expandable valve	325 (33.6)	225 (38.1)	44 (45.8)	74 (44.6)	0.006
Procedural time, min	36 (29–46)	36 (28–48)	38 (29–51)	37 (30–49)	0.526
Contrast agent, ml	85 (65–120)	85 (65–120)	90 (70–118)	95 (70–120)	0.222
Device success	851 (88.0)	520 (88.0)	86 (88.7)	145 (87.3)	0.991
MPG at discharge, mmHg	10 (8–13)	9 (6–12)	9 (7–12)	8 (6–11)	<0.001
Residual aortic regurgitation ≥ II°	26 (2.9)	24 (4.3)	2 (2.2)	6 (3.9)	0.442

Data shown as number (%) or median value (interquartal range). **Abbreviations:** HFpEF: heart failure with preserved ejection fraction; HFmrEF: heart failure with mid-range ejection fraction; HFrEF: heart failure with reduced ejection fraction; MPG = transvalvular mean pressure gradient

### Clinical outcomes

**Fig 2:**
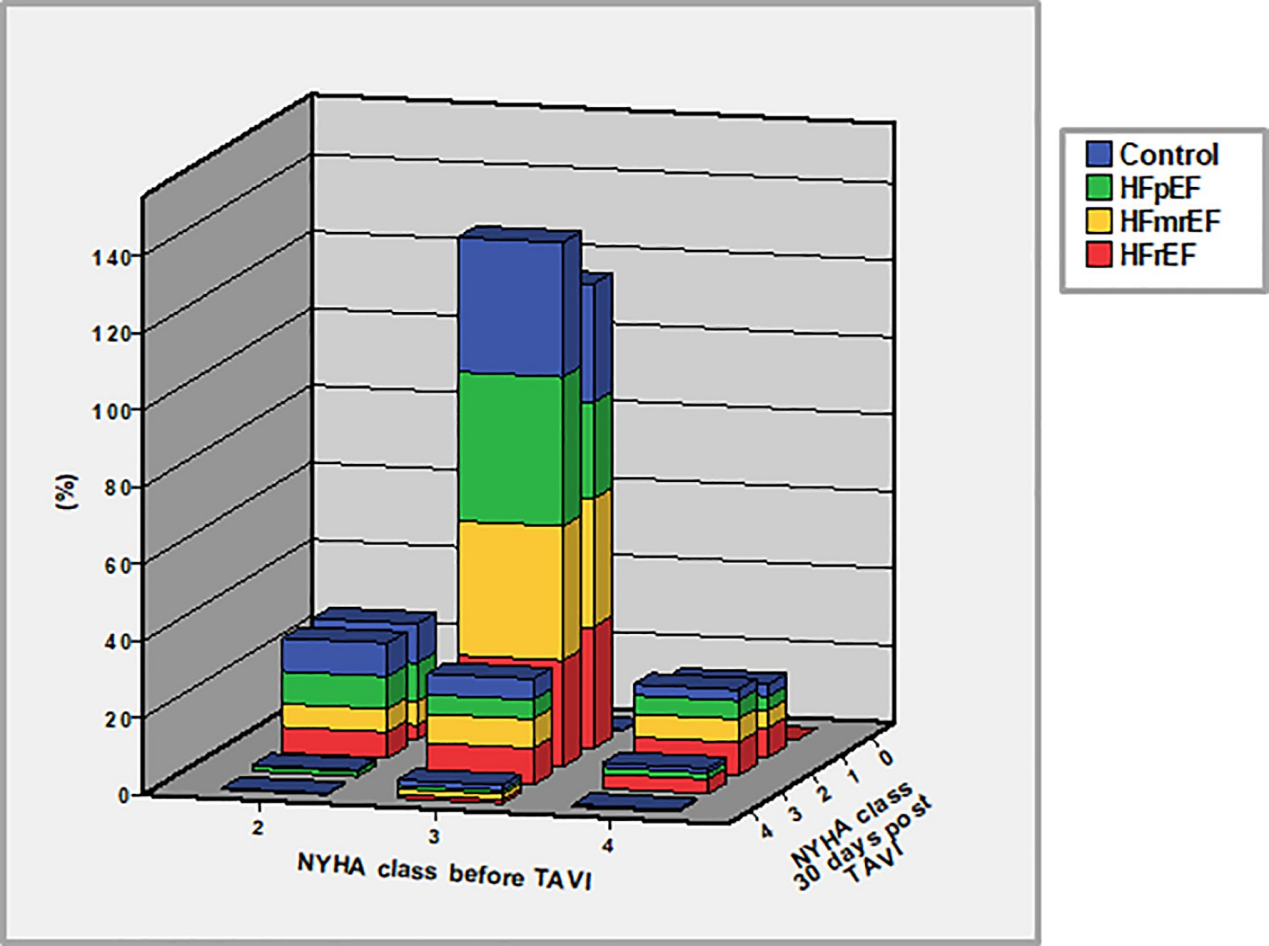
Change in NYHA functional status after TAVI. Changes in NYHA functional status from the timepoint before TAVI to the 30-day follow-up. Values are total percentages within each individual group.

Thirty-day overall mortality varied between 3.2% in controls and up to 6.6% in patients with HFrEF (p = 0.201) (Table 4). The relative in-hospital mortality and cardiovascular 30-day mortality were not different between groups. There were also no differences with respect to VARC-defined events during 30-day follow-up. Fourteen patients (8.5%) of the HFrEF group were implanted with an ICD. Functional NYHA status improved in >82% of all patients. Most patients demonstrated an improvement from NYHA class III to class II, and there were no differences in this improvement among groups (Fig 2). Interestingly, the percentage of patients showing an improvement from NYHA class III to class I was highest among HFrEF patients.

**Table 4:** Clinical outcomes.

	Control	HFpEF	HFmrEF	HFrEF	p
	n = 968	n = 591	n = 97	n = 166	
**30-Day Clinical Outcomes**					
Overall mortality	31 (3.2)	23 (3.9)	4 (4.1)	11 (6.6)	0.201
- in-hospital mortality	24 (77.4)	15 (65.2)	4 (100)	6 (54.5)	0.253
- cardiovascular mortality	28 (90.3)	17 (73.9)	2 (50.0)	9 (81.2)	0.169
Major stroke	5 (0.5)	3 (0.5)	1 (1.0)	0 (0)	0.702
Major vascular complication	72 (7.5)	62 (10.5)	10 (10.4)	15 (9.1)	0.194
New PM / ICD implant	150 (15.5) / 3 (0.3)	75 (12.7) / 0	17 (17.7) / 0	12 (7.3) / 14 (8.5)	<0.001
Acute kidney injury					
- Stage 1	24 (2.5)	17 (2.9)	4 (4.2)	8 (4.8)	
- Stage 2	12 (1.2)	12 (2.0)	2 (2.1)	2 (1.2)	0.509
- Stage 3	16 (1.7)	12 (2.0)	2 (2.1)	6 (3.6)	
Any VARC event	307 (31.7)	195 (33.1)	31 (32.3)	63 (38.2)	0.443
EF at 30 days					
- improved > 10%	62/618 (10.0)	45/360 (12.5)	25/53 (47.2)	48/92 (52.2)	
- unchanged	491/618 (79.4)	276/360 (76.7)	25/53 (47.2)	40/92 (43.5)	<0.001
- deteriorated < 10%	65/618 (10.5)	39/360 (10.8)	3/53 (5.7)	4/92 (4.3)	
NYHA at 30 days					
- improved	582/706 (82.4)	349/418 (83.5)	56/66 (84.8)	87/106 (82.1)	
- unchanged	106/706 (15.0)	59/418 (14.1)	9/66 (13.6)	17/106 (16.0)	0.275
- deteriorated	18/706 (2.5)	10/418 (2.4)	1/66 (1.5)	2 /106(1.9)	
**1-Year Clinical Outcomes**					
Overall mortality	99 (10.2)	80 (13.5)	13 (13.4)	39 (23.5)	<0.001
- cardiovascular mortality	70 (70.7)	52 (65.0)	8 (61.5)	28 (71.8)	0.762
Major stroke	10 (1.0)	5 (0.8)	1 (1.0)	0 (0)	0.621
New PM / ICD implant	161 (16.6) / 3 (0.3)	78 (13.2) / 0	18 (18.6) / 1 (1.0)	14 (8.4) / 17 (10.2)	<0.001
Decompensation after 30-day follow-up	22 (2.3)	17 (2.9)	2 (2.1)	5 (3.0)	0.851

Data shown as number (%). **Abbreviations:** HFpEF: heart failure with preserved ejection fraction; HFmrEF: heart failure with mid-range ejection fraction; HFrEF: heart failure with reduced ejection fraction; PM/ICD = pacemaker/implantable cardioverter-defibrillator; VARC = Valve Academic Research Consortium-2

**Fig 3:**
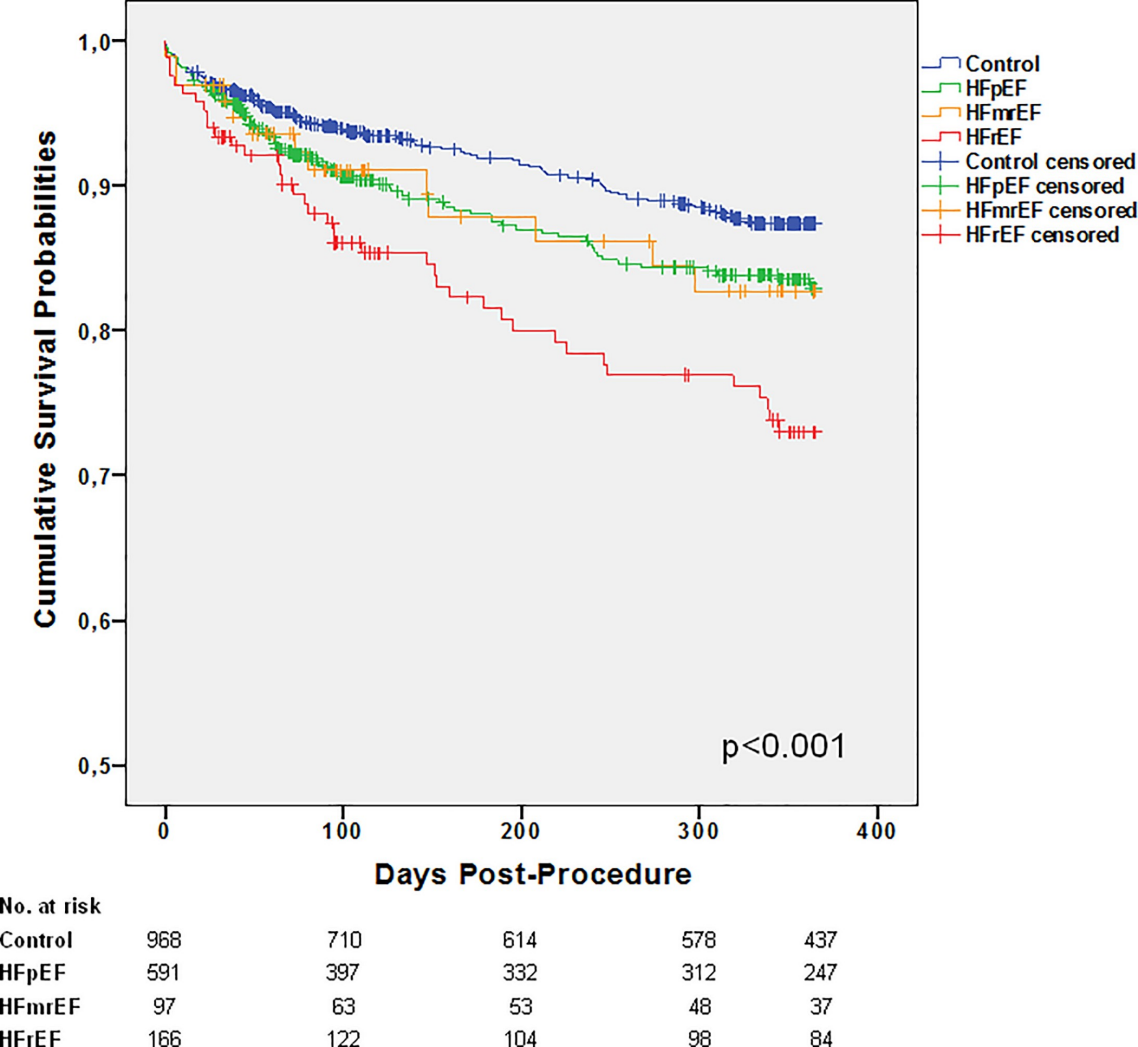
Survival curves based on all-cause mortality. Kaplan-Meier analysis of all-cause mortality of control patients and patients with HFpEF (heart failure with preserved ejection fraction), HFmrEF (heart failure with mid-range ejection fraction) and HFrEF (heart failure with reduced ejection fraction).

The median follow-up time was 350 (95–365), 335 (77–365), 297 (73–365), and 365 (96–365) days for patients in the control, HFpEF, HFmrEF, and HFrEF groups, respectively. Kaplan-Meier survival curves (Fig 3) showed that one-year survival was identical for patients with HFpEF and HFmrEF, with mortality rates of 13.5% and 13.4%, respectively. In contrast, patients with HFrEF had a mortality rate of 23.5%. Patients in the control group demonstrated a mortality rate of 10.2%. Survival curves were unchanged when HF patients were only included if they met the postulated criteria for the diagnosis of HF [21], i.e. all patients with EF <40% and all other HF patients with LVH (one-year mortality 13.4%, 10.1%, and 23.5% for HFpEF, HFmrEF, and HFrEF, respectively; p<0.001). Likewise, inclusion only of patients with positive criteria of severe AVC or AVCd also did not change the survival results. Whereas the changes in EF between baseline and 30-day follow-up had no influence on mortality in either of the groups, functional improvement early after TAVI was associated with a lower one-year mortality rate in the overall study population. Thus, patients who experienced a deterioration, no change, or an improvement in one or two NYHA classes at 30-day follow-up had one-year mortality rates of 25.8%, 9.9%, 6.2% or 4.6%, respectively (p<0.001). The cumulative numbers of ICD implantations (before TAVI plus during the one-year period after TAVI) in the different patient classes were 15 (1.5%), 2 (0.3), 4 (4.1%), and 36 (21.7) for controls and patients with HFpEF, HFmrEF, and HFrEF, respectively (p<0.001). Mortality rates in HFrEF patients with or without an ICD were 19.4% or 24.6%, respectively (p = 0.517).

### Predictors of mortality

In multivariate analysis (Table 5), a low body mass index (BMI), the presence of chronic obstructive pulmonary disease (COPD), a high STS score, a low SVI, a low MPG, and increasing systolic pulmonary artery pressures (SPAP) emerged as independent predictors of mortality in the overall population.

**Table 5:** Multivariate Cox regression analyses with mortality as outcome variable.

Variable	HR	CI 95%		p
		Lower	Upper	
BMI	0.949	0.916	0.985	0.005
COPD	1.591	1.090	2.323	0.016
STS-score	1.064	1.025	1.104	0.001
SVI	0.981	0.964	0.999	0.034
MPG	0.988	0.977	0.999	0.027
SPAP	1.010	1.000	1.021	0.044

**Abbreviations:** HR = hazard ratio, CI = confidence interval, BMI = body mass index, COPD = chronic obstructive pulmonary disease, STS = Society of Thoracic Surgeons, SVI = stroke volume index, MPG = transvalvular mean pressure gradient, SPAP = systolic pulmonary artery pressure

## Discussion

We determined that 1) patients with HFmrEF revealed a number of similarities with HFpEF patients but were characterized overall by a distinct, intermediate state; 2) TAVI caused a relief of symptoms in the majority of all HF patients; 3) periprocedural safety and early adverse events were not different in HF patients compared with controls; 4) the prognosis of patients with HFmrEF or HFpEF was almost as favorable as that of controls; and 5) one-year mortality in patients with HFrEF after TAVI was still considerably higher than in other HF patients.

### Heart failure in patients with symptomatic AS

The observation that correction of increased afterload is still associated with high mortality in certain patients post-TAVI has shifted the focus from the aortic valve alone to inclusion of the left ventricular myocardium as therapeutic target. Indeed, the concept of ventricular hemodynamic unloading by TAVI to improve LV function and HF symptoms in addition to optimal HF therapy will be tested in patients with moderate AS and HFrEF in another study [22]. Our patients demonstrated essential characteristics of HF such as impaired clinical status (worse NYHA functional status, higher rates of prior decompensations or impaired kidney function including the need for dialysis), echocardiographic markers (the presence of LVH and atrioventricular valve regurgitation), and biochemical markers (increasing levels of NT-proBNP). Indeed, these parameters not only mirror the weakened overall clinical status of our patients with concomitant HF but also represent cornerstones in the diagnostic algorithm for HF in current guidelines [21].

### Characteristics and prognosis in patients with HFmrEF

The key characteristic that differentiates patients with HFmrEF from other HF patients is an EF range of absolute 10 percentage points. This rather narrow range makes the diagnose of HFmrEF prone to errors, given that EF in our study was estimated visually. However, patients with HFmrEF in our study presented with distinct intermediate characteristics but showed similarities to patients with HFrEF with respect to the ischemic etiology of HF and the prevalence of concomitant coronary artery bypass surgery. The same observations were made for the Swedish Heart Failure Registry [23]. Here, patients with HFmrEF had an intermediate phenotype and HFmrEF and HFpEF patients had a similar prognosis after adjustment for age and other confounders at post-procedural time points up to 3 years that was better than in patients with HFrEF. In contrast, the results of another large patient cohort showed that all-cause mortality in patients with HFmrEF was the same as in those with HFrEF [24]. Furthermore, two recent meta-analyses report that patients with HFmrEF had a lower mortality than those with HFrEF or HFpEF [25] [26], which is in clear contrast to our results. In summary, data available on patients with HFmrEF are conflicting, and it is still difficult to draw general conclusions concerning characteristics and outcome in patients with this phenotype, potentially due to the wide spectrum of patients who may be included in this category (e.g. acute vs. stable, new-onset vs. chronic) [27] and due to an uncertainty about treatment targets [28].

### Survival in HFrEF patients

Patients with HFrEF revealed the highest mortality compared with other HF patients. Mortality during 30-day follow-up was already markedly higher than in both other groups and these differences were highly significant after a one-year follow-up. On one hand, these differences in mortality could be explained by the higher overall cardiovascular risk of HFrEF patients, mirrored by a higher STS score. On the other hand, one could speculate that medical HF treatment, which has been demonstrated to have tremendous prognostic impact in these patients, was lacking. Indeed, one-year mortality in those of our HFrEF patients who had NYHA status II/III at 30 days after TAVI was still 16.7%, and the same mortality rate (15%) was described in NYHA classes II/III in a study dating from 1987 when patients with HFrEF were not yet prescribed ACE inhibitors [29]. Furthermore, an adjusted overall risk reduction by 41% of cardiovascular mortality at the 3-year follow-up post-TAVI by renin-angiotensin system inhibitors may also corroborate the importance of this therapy for cardiovascular protection [30]. One can speculate that these parallels support the concept of concomitant HF as the main driver of mortality in patients with AS, even after TAVI. This idea is also consistent with the tendency for lower mortality in patients with an ICD compared with those without. Unfortunately, all further analysis of the prognosis in our patients with HFrEF is limited due to the grave lack of information on HF therapy. Furthermore, one can only speculate about the outcome of our HFrEF patients if they had not undergone TAVI. Nevertheless, it is interesting to see that survivors in the HFrEF group revealed at least the same clinical benefit after TAVI (mirrored by an improvement in NYHA functional class) as other HF patients and even controls.

### Predictors of mortality

In prior analyses [31], a higher BMI before TAVI was associated with lower mortality in accordance with the “obesity paradox” described in patients undergoing open-heart surgery. A high BMI also emerged as an independent predictor of survival in the present study. It is interesting to note that the presence of COPD and SPAP were also predictors of mortality, as both diseases are clearly related to right- and left heart failure and corroborate the significance of concomitant HF in this study [32] [33]. The negative predictive value of a low SVI and low MPG for mortality has been repeatedly demonstrated in earlier studies [5, 7].

### Limitations

Echocardiographic measurements were made by different operators without a centralized core lab. SVI was determined by Doppler echocardiography, which implies angle-dependent errors. EF was estimated visually, which mirrors the everyday reality of this all-comers registry. We had no information on LV diastolic function in our patients. A complete dataset of natriuretic peptide levels and information on HF medication would have added great value to our results.

## Conclusions

TAVI can be performed safely in patients with AS and low SVI who meet the criteria for concomitant HF and is associated with substantial clinical benefits. Although 30-day mortality and adverse events were not different among controls and patients of different HF classes, and one-year survival in HFmrEF and HFpEF patients was as favorable as that of controls, HFrEF patients still had a high one-year mortality. Ventricular unloading by TAVI appears to benefit prognosis in HFmrEF and HFpEF patients, whereas its effects are less impressive once systolic LV function has significantly failed in HFrEF patients.

## Supporting information

S1 Text Ethical approval of this study (German version). (PDF)
