# Supplementary material for: Outcome of patients with heart failure after transcatheter aortic valve implantation
Source: PLoS One. 2019 Nov 26;14(11):e0225473. doi: 10.1371/journal.pone.0225473 (PMC6879149; doi:10.1371/journal.pone.0225473)
Supplement: S1 Text — (PDF) [file pone.0225473.s001.pdf]

JUSTUS-LIEBIG
UNIVERSITÄT
GIESSEN

FACHBEREICH 11
MEDIZIN

ETHIK-KOMMISSION
des FB Medizin

Vorsitzender: Prof. Dr. H. Tillmanns

Klinikstr. 29 (Alte Chirurgie)
D -35385 Gießen

Tel.: (0641)99-42470 / 47660
Fax: (0641)99-42479
E-Mail: ethik.kommission@pharma.med.uni-giessen.de

Ethik-Kommission, Klinikstr. 29 (Alte Chirurgie), D-35385 Gießen

PD Dr. Fischer-Rasokat
Kerckhoff-Klinik Bad Nauheim
Kardiologie
Benekestr. 2-8
61231 Bad Nauheim

Gießen, den 20. Juli 2018
Dr. Kr./

Votum der Ethik-Kommission AZ 145/18

Sehr geehrter Herr PD Dr. Fischer-Rasokat,

das Projekt (AZ **145/18:** *Outcome of patients with heart failure and mid-range ejection fraction after transcatheter aortic valve implantation (TAVI).)* wurde außerhalb der Sitzung der Ethikkommission begutachtet.

Hintergrund:
Es handelt sich um die retrospektive Analyse der Daten aller Patienten bis etwa 2017 mit durchgeführter TAVI wegen einer eingangs vorliegenden Herzinsuffizienz mit mittelgradig eingeschränkter Ejektionsfraktion, mit dem Ziel eines Vergleichs von Basischarakteristika und 1-Jahres-Überlebenskurven mit denen anderer TAVI-Patienten. Als Datenquelle dient die kardiologische Biodatenbank für Herzkrankheiten der Kerckhoff-Klinik Bad Nauheim und des UKGM Gießen und Marburg, Standort Gießen (AZ der Ethikkommission 199/15), in welche prospektiv seit 2010 alle Patienten nach TAVI eingeschlossen werden (Unterregister BioValve, AZ der Ethikkommission 99/13, bislang etwa 2500 Patienten). Alle Patienten werden zu hausinternen Qualitätskontrollen nach 3 Monaten und 1 Jahr in die Ambulanz einbestellt oder telefonisch kontaktiert, um follow-up-Daten zu erfragen (Überleben, CV-Ereignisse, NYHA-Stadium); diese follow-up-Daten gehen in die Registerdatenbank mit ein.

Hauptzielgrößen sind die Überlebensrate [Gesamtmortalität und kardiovaskuläre (CV)-Mortalität]. Weitere Zielgrößen sind nicht vorgesehen.

Die erwarteten Gruppengrößen liegen zwischen 100 und 600 Patienten pro Gruppe, je nach Charakterisierung der einzelnen Subgruppen.

Analysen und Methodik:
Nicht-parametrischer Vergleich von Gruppen, Kaplan-Meier-Überlebenskurven, Cox-Regressions-Analysen, propensity-score matching.

Die Patienten wurden über die Verwendung ihrer Daten zu Forschungszwecken aufgeklärt und haben zugestimmt.
Die Ethikkommission hat sowohl das Unterregister BioValve wie auch die zusammengefasste Biodatenbank positiv bewertet.

Die Kommission stimmt der Durchführung der klinisch wichtigen Datenauswertung nach Art einer Qualitätskontrolle ohne Einwände zu und wünscht gutes Gelingen.

Mit freundlichen Grüßen

Prof. Dr. H. Tillmanns
Vorsitzender der Ethik-Kommission
